# Concerns with the revised Japanese recommendation for administering vitamin C to septic patients

**DOI:** 10.1186/s40560-023-00702-2

**Published:** 2023-11-13

**Authors:** Harri Hemilä, Elizabeth Chalker

**Affiliations:** 1 Department of Public Health, University of Helsinki, P.O. Box 20, 00014 Helsinki, FI Finland; 2 Biological Data Science Institute, Australian National University, Canberra, ACT 2601 Australia

**Keywords:** Critical care, Guidelines, Intensive care units, Meta-analysis, Mortality, Randomized controlled trials, Treatment outcome

To the Editor,

In the revision of the recommendation for administering vitamin C for sepsis patients, Egi and Ogura write *“the Japanese Clinical Practice Guidelines for Management of Sepsis and Septic Shock 2020… suggested administering vitamin C to septic patients”* [1]. They carried out a new literature search and identified 12 new RCTs, performing a revised meta-analysis of 23 RCTs in all and reversing the 2020 recommendation [1]. However, 13 of the 23 listed trials administered combinations of antioxidants and other drugs, such as hydrocortisone and thiamine [2]. If the scientific question is about the specific effect of vitamin C on sepsis, then the included trials should only examine vitamin C. For example, a recent observational study indicated significantly different effects from vitamin C alone and from vitamin C together with hydrocortisone [3].

Egi and Ogura write *“Lamontagne *et al*.* [4] *conducted a large multicenter RCT* [the LOVIT trial]*, … This RCT revealed that the proportion of a composite of death or persistent organ dysfunction at 28 days in the vitamin C group was significantly higher than that in the placebo group.”* However, they disregard the fact that there was no difference between the vitamin C and placebo groups when vitamin C was being administered. The difference between the groups occurred after vitamin C was abruptly stopped [5]. Furthermore, the harm in the vitamin C group rapidly diminished over time after the termination of the vitamin, indicating that the time of termination was relevant. Abruptly ceasing ongoing vitamin C administration may cause a rebound effect which has been observed empirically, in which vitamin C levels can decline to levels that are lower than those before the supplementation started [6–9]. Thus, it seems plausible that the harm observed after stopping vitamin C in the LOVIT trial is explained by the rebound effect [5].

They further continue: *“… long-term mortality was chosen as the effect on mortality since we predetermined that the highest certainty of evidence was adopted.”* We will all suffer from long term mortality eventually, so it is not an ideal outcome for short term acute conditions. Long-term mortality is less relevant than short-term effects at the time of the acute illness. For example, in the CITRIS–ALI trial, mortality reduced dramatically when vitamin C administration was ongoing, but the trial groups did not differ thereafter [10]. The late follow-up dilutes the effects observed around the time of the acute stages of illness.

In our view, Egi and Ogura’s conclusions place too great a weight on “long-term mortality”. Other outcomes are also important even if they are not associated with mortality. For example, a meta-analysis of 12 trials with 1766 patients found that vitamin C alone reduced the length of ICU stay on average by 7.8% [11]. Another meta-analysis found that in five trials, including 471 patients; vitamin C alone shortened ventilation time on average by 25% [12]. Irrespective of long-term mortality, such outcomes are relevant in the hospital context. They are related to hospital costs and outcomes important for patients, even if eventually all patients end their hospital stay in good health.

The role of vitamin C for sepsis patients is not clear and warrants further study. However, based on the findings from studies of vitamin C alone [3, 10–12], there is no justification to firmly recommend *“against administering vitamin C to septic patients”* [1]. That conclusion was based on 23 trials, half of which did not administer vitamin C alone. Therefore, the revised meta-analysis is not valid as to the specific effects of vitamin C.

## Data Availability

Not applicable.
